# Nutrient connectivity via seabirds enhances dynamic measures of coral reef ecosystem function

**DOI:** 10.1371/journal.pbio.3003222

**Published:** 2025-07-08

**Authors:** Cassandra E. Benkwitt, Anna Zora, Ameer Ebrahim, Rodney Govinden, Ines D. Lange, Sean Evans, Melissa Schulze, Emma Cotton, Lucie Bennett, Nicholas A. J. Graham

**Affiliations:** 1 Lancaster Environment Centre, Lancaster University, Lancaster, United Kingdom; 2 Fregate Island Sanctuary, Fregate Island, Seychelles; 3 Ameer Ebrahim Consulting, Victoria, Mahé, Seychelles; 4 Seychelles Fishing Authority, Victoria, Mahé, Seychelles; 5 Geography, College of Life & Environmental Sciences, University of Exeter, Exeter, United Kingdom; 6 C/O Cousine Island Company Limited, Cousine Island, Providence, Mahé, Seychelles; 7 Island Conservation Society, Pointe Larue, Mahé, Seychelles; 8 Ramos Marine and Island Reserve, Félicité Island, Seychelles; Estacion Biologica de Doñana CSIC, SPAIN

## Abstract

Cross-ecosystem nutrient fluxes can influence recipient food webs, including both static measures of structure and dynamic measures of function. However, a mechanistic basis for how nutrient subsidies affect both structure and function across multiple trophic levels is still lacking. Here, we investigate how nutrient subsidies provided by seabirds influence coral reefs, focusing on the link between primary producers and primary consumers. We quantified turf algal cover and herbivorous fish biomass (static metrics of structure), as well as productivity of turf algae and herbivorous fish (dynamic metrics of function) at sites in the inner Seychelles with a range of seabird densities due to different rat invasion histories. Turf algae grew faster with increasing amounts of seabird-derived nutrients. These higher rates of primary productivity, in turn, fueled higher productivity and biomass of herbivorous fishes. In contrast, seabird-derived nutrients did not increase cover of turf algae nor did turf algal cover affect herbivores. Instead, seabird nutrients indirectly enhanced herbivorous fish productivity and biomass via effects on primary productivity, which, in turn, led to increased top-down control by herbivores to limit turf algal cover. Overall, dynamic metrics better revealed the flow and effects of seabird-derived nutrients through coral-reef food chains and revealed the mechanisms by which seabirds can enhance coral-reef ecosystem function. These findings could be used to predict the benefits of removing introduced rats from islands, which can increase seabird populations and restore nutrient connectivity, thus potentially enhancing ecosystem function across multiple trophic levels on coral reefs.

## Abstract 2

Cross-ecosystem nutrient fluxes can influence recipient food webs, including both static measures of structure and dynamic measures of function. This study of nutrient subsidies provided by seabirds on coral reefs shows that dynamic metrics better revealed the flow and effects of seabird-derived nutrients through coral-reef food chains, and allowed prediction of the benefits of removing introduced rats from islands.

## Introduction

Understanding the structure and function of ecosystems is a central goal of ecology. While structure can be observed relatively easily, underlying function (i.e., movement of energy and materials) is more difficult to quantify [1–3]. Despite the challenges, integrating both static measures of structure (e.g., standing crop, biomass) and dynamic measures of function (e.g., growth, productivity) is necessary to fully understand the patterns and underlying processes that define ecosystems [4]. Such knowledge is even more pressing in today’s rapidly changing world, as quantifying how disturbances influence both static and dynamic metrics can better inform conservation of ecosystems and their associated functions [5].

Ecosystems are connected to each other via the movement of nutrients, energy, materials, and animals, which can have a large influence on structure and function [6–8]. Because primary producers are often constrained by nutrient availability, nutrient inputs at the base of ecosystems can increase nutrient uptake by plants, in turn, boosting plant biomass and productivity and altering plant community composition. Such changes have been observed in multiple ecosystems, including on oceanic islands receiving marine-derived nutrient inputs from seabirds and in forests subsidized by salmon migrating up streams [9–12]. Nutrient subsidies can precipitate changes at higher trophic levels as well, including increased biomass of consumers on marine-subsidized islands [11,13–16] and enhanced invertebrate productivity in streams receiving forest litter fall [17,18]. However, most studies focus either on static metrics or on a single trophic level within recipient ecosystems, meaning an integrated understanding of how nutrient inputs influence both static and dynamic metrics across multiple trophic levels is lacking. Linking dynamic and static properties may provide a mechanistic basis for how ecosystem connectivity influences structure and function. As such, the implications of losing ecosystem connectivity, which is rapidly eroding due to human activities (e.g., invasive predators reducing seabird populations, dams preventing salmon migrations), would be more apparent.

Here, we use a causal modeling framework to test the effect of allochthonous nutrient inputs on static and dynamic measures of ecosystem structure and function across two trophic levels. Coral reefs provide an ideal setting for this study, as tight feedbacks between primary and secondary productivity have long been recognized, yet biomass can be de-coupled from productivity in both primary producers and consumers [19–21]. Therefore, a simultaneous focus on both static and dynamic metrics across trophic levels is particularly relevant. In addition, while coral reefs represent highly productive spatially coupled ecosystems, nutrient flows and their effects are still unresolved [3]. Moreover, many of these natural nutrient connections have been interrupted, creating relevant “natural experiments” in which to test the effects of ecosystem connectivity and its loss. For example, seabirds can transfer large amounts of nutrients from oceanic feeding grounds to nearshore coral reefs via the islands they nest and roost on, yet introduced predatory rats cause dramatic reductions in seabird populations [22,23]. Around rat-free islands, seabird populations can thrive and their nutrient subsidies can enhance biomass and productivity of herbivorous reef fishes [24–26]. However, whether seabird nutrients influence primary producer cover and productivity on coral reefs, and the mechanistic pathway by which herbivorous consumers are enhanced, remain unknown.

**Fig 1:**
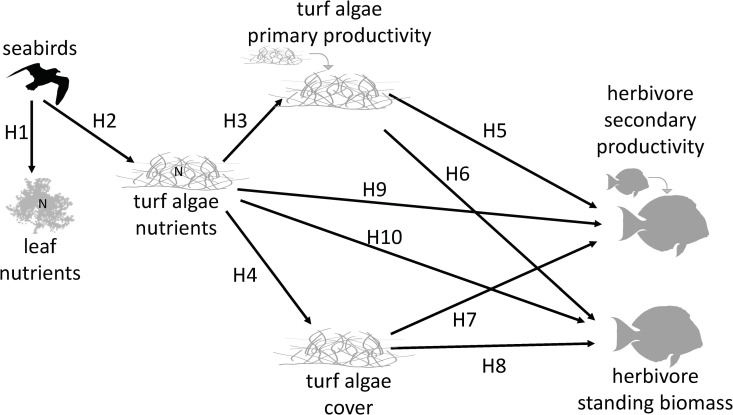
Conceptual overview of hypothesized bottom-up causal relationships within our study system. We tested the following *a priori* hypotheses: seabirds increase nutrients of coastal leaves (H1) and algal turfs (H2). Higher turf nutrients lead to faster turf growth (H3) and higher turf cover (H4). Faster turf growth leads to higher herbivore productivity (H5) and biomass (H6). Higher turf cover leads to higher herbivore productivity (H7) and biomass (H8). Higher turf nutrients lead to higher herbivore productivity (H9) and biomass (H10). For hypotheses H9–H10, turf nutrients could affect herbivores either directly (due to increased food quality, lines labeled H9, H10) and/or indirectly via their effects on turf growth (i.e., food replenishment, lines labeled H3–H5, H3–H6) or turf cover (i.e., food quantity, lines labeled H4–H7, H4–H8). Therefore, we compared the direct effect (food quality only) to the total effect (food quality + food quantity + food replenishment) of turf nutrients on herbivore biomass and productivity to elucidate the pathway(s) by which seabird nutrients in algal turfs influence consumers. These causal pathways were tested within broader directed acyclic graphs (DAGs), which included potential confounding variables (S1A Fig). Based on our initial findings, we built an alternative DAG in which H7 and H8 are reversed to test the hypothesis that herbivore productivity and biomass have negative effects on turf cover via top-down controls (S1B Fig).

To build an integrated understanding of how seabird-provided nutrients to coral reefs influence static measures of structure and dynamic measures of function across trophic levels, we test a series of *a priori* causal hypotheses (Figs 1 and S1). First, we establish whether seabird-provided nutrient subsidies are taken up by primary producers on both islands and adjacent coral reefs. We then test how seabird nutrient inputs influence algal turf cover (static metric) and primary productivity, measured as growth rate (dynamic metric). Next, we test how algal turf cover and productivity influence herbivorous fish biomass (static metric) and productivity (dynamic metric), and in turn, determine the pathway by which seabird nutrients affect herbivorous fishes (directly via enhanced primary producer nutrient content [i.e., food quality], or indirectly via cover [i.e., food quantity] and/or primary productivity [i.e., food replenishment]). Finally, we resolve the overall pathways of seabird-provided nutrient influence on this system by integrating their bottom-up effects with the top-down effects of herbivores (S1 Fig).

## Results

### Seabirds enhance nutrients in primary producers

**Fig 2:**
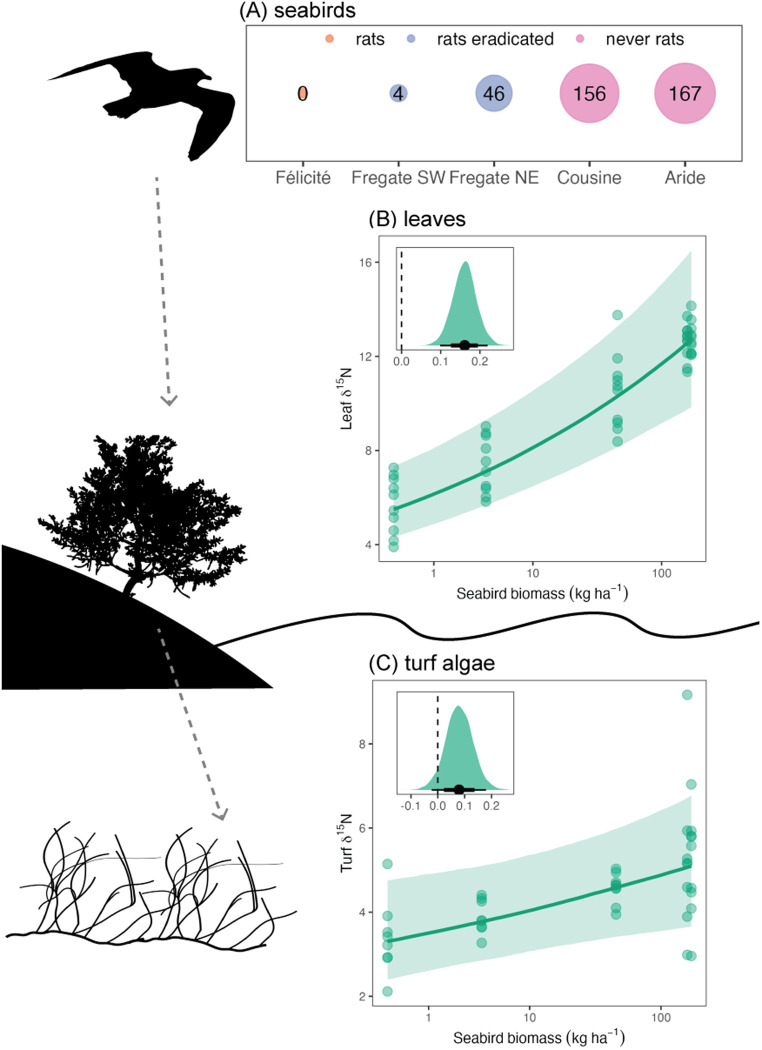
Effect of seabird biomass on nutrient uptake by terrestrial plants and marine turf algae. **(A)** Seabird biomass (kg/ha) across the five study sites as a function of rat invasion status. **(B, C)** Effect of seabird biomass on δ^15^N (a proxy for seabird-provided nutrients) in coastal plants (B) and marine turf algae (C). Main plots—points represent partial residuals from Bayesian models, lines represent estimated conditional effects, and shading represents 75% Bayesian credible intervals. Insets—posterior predictive distributions for effect of seabird biomass on response (both log-transformed). Point represents median estimate and lines represent 95% and 75% highest posterior density intervals (HPDIs). The data underlying this figure can be found in https://doi.org/10.5281/zenodo.15485420.

Breeding seabird biomass ranged from 0 to 170 kg/ha adjacent to the five marine study sites across four islands in the inner Seychelles, with the relative biomass corresponding to whether invasive rats were present, eradicated, or always absent from the islands (Figs 2A and S2 and S1 and S2 Tables). Where present, seabirds inputted nutrients into the system, which were assimilated by terrestrial coastal plants and, subsequently, turf algae at nearby marine sites (Fig 1H1-H2). This nutrient pathway is evidenced by an increase in δ^15^N, which is an indicator of seabird-derived nutrients, by a factor of 1.12 in leaves and 1.06 in turf algae with each doubling of seabird biomass (Fig 2B and 2C; leaves 95% highest posterior density interval [HPDI] = 1.07 to 1.16, posterior probability of expected effect [PP] > 0.99; turf 95% HPDI = 0.98–1.13, PP = 0.94).

### Seabird-derived nutrients increase marine primary productivity but not turf cover

**Fig 3:**
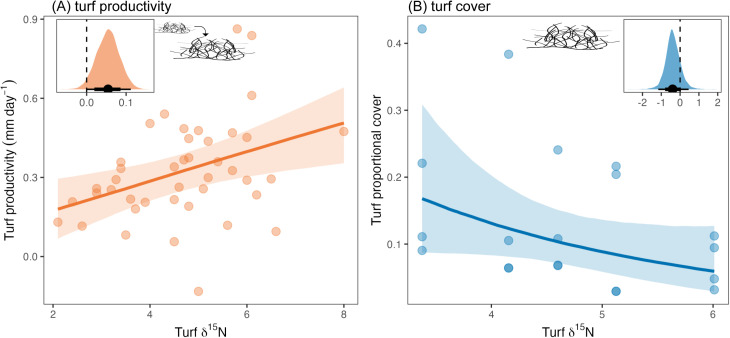
Effect of seabird nutrients in turf algae on (A) turf algal productivity and (B) turf algal cover. Main plots—Points represent partial residuals from Bayesian models, lines represent estimated conditional effects, and shading represents 75% Bayesian credible intervals. Insets—Posterior predictive distributions for effect of seabird nutrients in turf on log-transformed turf growth (A) or turf cover (B). Point represents median estimate and lines represent 95% and 75% highest posterior density intervals (HPDIs). The data underlying this figure can be found in https://doi.org/10.5281/zenodo.15485420.

To determine the effects of this seabird-derived nutrient pathway on primary producers, we measured turf algal growth within herbivore exclusion cages (dynamic metric) and turf algal cover along benthic transects (static metric) (Fig 1 H3-H4). Seabird nutrients, in turn, increased marine primary productivity. For each one unit increase in turf δ^15^N, turf algae grew a total of 0.05 mm day^−1^ faster (Fig 3A; 95% HPDI = 0.00–0.11, PP = 0.97). After accounting for initial turf height, the direct effects of seabird nutrients on turf growth were similar to their total effects (estimate = 0.04, 95% HPDI = −0.01–0.10, PP = 0.94), and the effects of seabird nutrients on primary productivity were also similar when using proportional turf growth, rather than absolute growth, as the response (estimate = 0.03, 95% HPDI = 0.00–0.07, PP = 0.98). By contrast, there was no evidence for the hypothesized positive effect of seabird nutrients on turf cover, with a 32.9% reduction in turf cover for each one unit increase in turf δ^15^N (Fig 3B; 95% HPDI = −77.5% to 38.2%, PP = 0.15). Thus, for primary producers, the dynamic, but not static, metric responded positively to seabird nutrients.

### Seabird-derived nutrients indirectly increase herbivore biomass and productivity via increased primary productivity

Moving up in trophic level, we tested whether turf algal growth, cover, or nutrients had positive bottom-up effects on herbivorous fishes. Herbivorous fishes were censused along the same transects as algal turf cover, with standing biomass estimated using published species-specific Bayesian length–weight relationships (static metric) and productivity calculated using predicted growth trajectories (dynamic metric).

**Fig 4:**
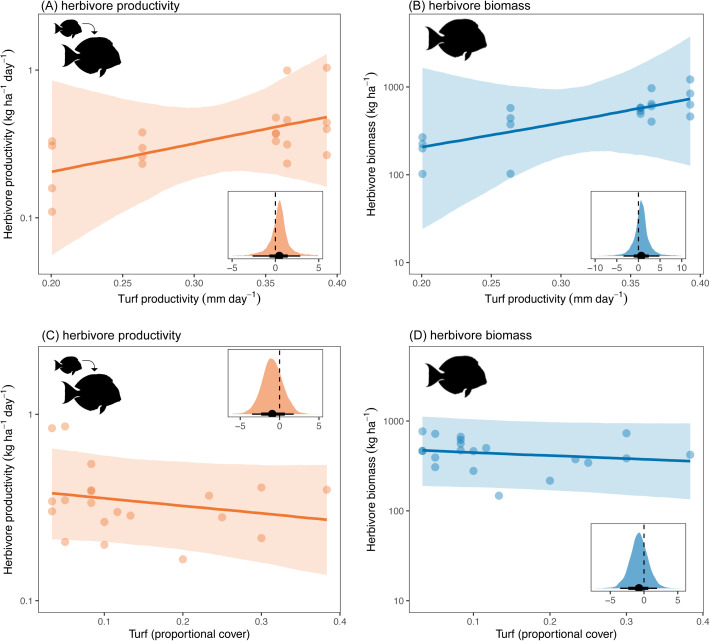
Effect of turf productivity on (A) herbivore productivity and (B) herbivore biomass, and effect of turf cover on (C) herbivore productivity and (D) herbivore biomass. Main plots—Points represent partial residuals from Bayesian models, lines represent estimated conditional effects, and shading represents 75% Bayesian credible intervals. Insets—Posterior predictive distributions for effect on log-transformed response. Point represents median estimate, and lines represent 95% and 75% highest posterior density intervals (HPDIs). The data underlying this figure can be found in https://doi.org/10.5281/zenodo.15485420.

Primary productivity caused increases in both herbivore productivity and standing biomass (Fig 1 H5H6). For each additional 0.1 mm day^−1^ of turf growth, herbivore productivity increased by a factor of 1.57 and herbivore biomass increased by a factor of 1.94, although there were wide uncertainty intervals around these estimates (Fig 4A and 4B; productivity 95% HPDI = 0.00–12.37, PP = 0.71; biomass 95% HPDI = 0.00–44.00, PP = 0.70). By contrast, there was no evidence that the static metric of turf cover enhanced herbivore productivity or biomass (Figs 1 H7-H8 and 4C, and 4D; estimated multiplicative effect on productivity = 0.91, 95% HPDI = 0.68–1.17, PP = 0.24; biomass = 0.93, 95% HPDI = 0.69–1.19, PP = 0.27).

**Fig 5:**
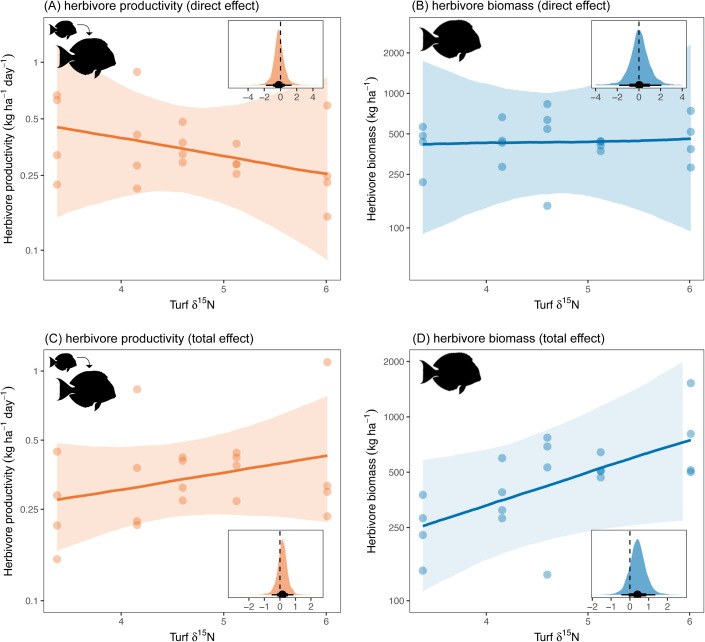
Direct and total effect of turf nutrients on herbivore growth and herbivore biomass. Direct effects are driven by food quality (turf nutrients) alone, while total effects also include indirect pathways via food replenishment (turf productivity) and food availability (turf cover). Main plots—Points represent partial residuals, lines represent estimated conditional effects, and shading represents 75% Bayesian credible intervals. Insets—Posterior predictive distributions for effect on log-transformed response. Point represents median estimate and lines represent 95% and 75% highest posterior density intervals (HPDIs). The data underlying this figure can be found in https://doi.org/10.5281/zenodo.15485420.

Because the amount of seabird-derived nutrients in turf algae have the potential to benefit consumers via multiple pathways (Fig 1), we compared the direct versus total effects of algal nutrients on herbivore biomass and productivity. Direct effects would be only via increased turf quality (Fig 1 H9-H10), whereas total effects would be via their effects on turf quality plus turf growth (Fig 1 H3 and H5–H6) and turf cover (Fig 1 H4 and H7–H8). The proportion of seabird-derived nutrients in turf algae had limited direct effects on herbivore biomass and productivity (Fig 5A and 5B, estimated multiplicative effect on biomass = 1.03, 95% HPDI = 0.00–4.95, PP = 0.52; productivity = 0.81, 95% HPDI = 0.03–2.79, PP = 0.35). However, when examining the total effect, which includes both direct effects (i.e., food quality) and indirect effects (via food replenishment and/or quantity), seabird-provided nutrients in turf had a positive influence on herbivores. For each one-unit increase in turf δ^15^N, there was an estimated increase in herbivore biomass and productivity by a factor of 1.50 and 1.17, respectively (Fig 5C and 5D, biomass 95% HPDI = 0.41–3.24, PP = 0.85; productivity 95% HPDI = 0.46–2.14, PP = 0.72). Combined with the result that seabird nutrients enhanced turf productivity but not turf cover, these findings indicate that higher seabird-derived nutrients in turf algae indirectly increased herbivore biomass and productivity via their positive effects on primary productivity (i.e., food replenishment).

**Fig 6:**
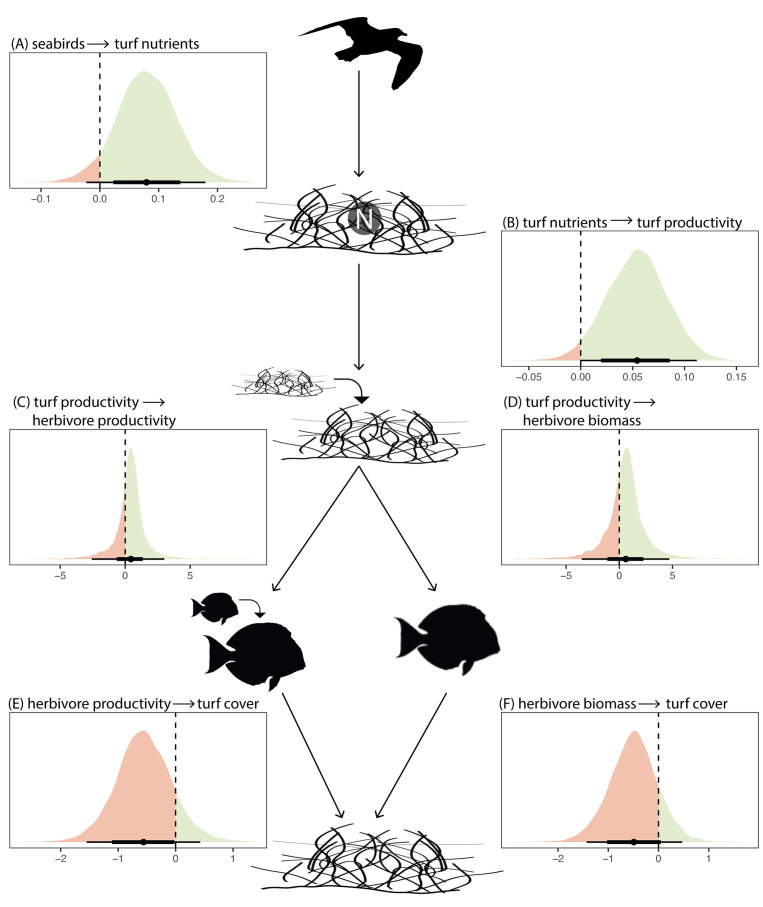
Causal pathways of seabird nutrient inputs on turf algae and herbivorous fishes. Results are from Bayesian models testing for causal pathways based on an alternative DAG assuming both bottom-up and top-down controls in this system (S1B Fig). **(A–F)** Posterior predictive distributions for each causal pathway, point represents median estimate and lines represent 95% and 75% HPDIs. Distributions are colored to show positive effects in green and negative effects in red. The data underlying this figure can be found in https://doi.org/10.5281/zenodo.15485420.

### Seabird-derived nutrients stimulate top-down control of turf algal cover

Thus far, we have focused on the bottom-up effects of seabird-derived nutrients on algal-herbivore dynamics based on *a priori* hypotheses of how allochthonous nutrient inputs influence structure and function in recipient systems. However, herbivores can also have top-down effects on primary producers, so we constructed a second DAG in which we test for this causal pathway (S1B Fig). We still assume bottom-up effects of seabird nutrients on primary productivity, and of primary productivity on herbivores because our above results provide evidence for these pathways and because primary productivity was measured in herbivore exclusion cages. Under these assumptions, herbivores negatively affected turf cover—for each doubling of herbivore productivity, turf cover decreased by 32.12% (95% HPDI = −70.73% to 24.66%, PP = 0.88) and for each doubling of herbivore biomass, turf cover decreased by 28.57% (95% HPDI = −69.25% to 28.29%, PP = 0.85). Consistent with our initial results, there were still positive bottom-up effects of seabird nutrients on turf productivity, and of turf productivity on herbivore biomass and productivity (Fig 6). This combination of bottom-up and top-down effects, with seabird nutrients stimulating herbivore biomass and productivity via increased primary productivity, and herbivores, in turn, reducing turf algal cover (Fig 6), explains the observed negative total effect of seabird nutrients on turf cover (Fig 3B).

Several correlative (non-causal) analyses provide further support for these pathways of seabird nutrient effects. First, turf nutrients, turf productivity, herbivore biomass, and herbivore productivity were all positively correlated, while all of these responses were negatively correlated with turf cover (S4 Fig). Multivariate analyses of herbivorous fish functional groups also provide support for the prediction that, if turf algal nutrients and turf productivity have bottom-up effects on herbivores, then herbivores that specifically feed on turf algae should be most positively associated with these variables. Turf-feeding croppers had the strongest positive associations with turf nutrients and turf productivity, while farming damselfish (that maintain their own territories of algal turfs) had the strongest negative correlations (S5 Fig). Overall, turf productivity, turf nutrients, and structural complexity showed the strongest associations with herbivorous fish communities by feeding group. Finally, we conducted a multivariate analysis of benthic communities to test the prediction that, if turf cover is controlled by herbivores, then turf cover should be most negatively associated with herbivorous fish functional groups that remove turf algae. Indeed, turf algae and sand/rubble were strongly negatively correlated with biomass of herbivorous fish that function to remove turf algae, which include those groups that specifically target turf algae (e.g., croppers), as well as groups that may not target turf algae but still remove it due to their feeding location and mode (e.g., sediment suckers, scrapers, excavators). In addition to biomass of turf-removing herbivores, turf nutrients, structure, and exposure were also associated with benthic community structure (S5 Fig).

## Discussion

Allochthonous nutrients have the potential to drive structure and function in recipient ecosystems. Here, we show that cross-ecosystem nutrient inputs stimulate dynamic measures of function, but not static metrics of structure, of an important primary producer on coral reefs. Similarly, algal turf productivity, but not algal turf cover, enhanced both productivity and standing biomass of herbivorous consumers. Nutrient subsidies had no direct effect on herbivorous fishes (e.g., via increased food quality); instead, they stimulated consumers solely via indirect benefits to primary productivity. Using causal modeling and integrating both static and dynamic metrics across multiple trophic levels provided new insights into the role of cross-ecosystem nutrient subsidies on recipient ecosystems, and identified mechanisms for higher productivity of consumers.

Algal turfs are a key component of coral reef ecosystems [27,28]. Hence, understanding coral reef structure and function necessitates an understanding of algal turf cover and productivity. We provide, to our knowledge, the first estimates of turf productivity for the Indian Ocean. Based on modeled turf growth rates from [28], our estimates are at the high end of the expected range on natural substrates. The high observed rates may be, in part, due to our use of sites at approximately 4–5 m depth, as turf productivity remains consistently high down to approximately 5 m, before decreasing at greater depths [28]. However, all seabird sites had turf growth rates greater than the predicted mean of 0.19 mm/day, while the reef near the rat-infested island had turf growth rates approximately equal to the mean [28]. Combined with the fact that introduced rats are widespread across the world’s tropical islands [29], these findings beg the question of whether coral reef benthic productivity has been reduced adjacent to islands worldwide by the presence of invasive rats and disruption of natural nutrient subsidies? Given that primary production and herbivory are two of eight core processes for coral-reef ecosystem functioning [30], restoring lost nutrient flows and boosting primary productivity may be one facet of restoring reef functions.

The role of algal turfs is evolving, as they are increasingly implicated in benthic shifts from coral-dominated to algal-dominated reefs [31,32], and are rewiring trophic pathways on tropicalized temperate reefs [33]. Although human-caused nutrient addition can promote turf dominance [34], we observed that seabird nutrients did not increase turf cover. Instead, seabird nutrients appeared to increase top-down controls that help constrain turf coverage—by increasing primary productivity, seabird nutrients increased herbivore biomass and productivity, which, in turn, consume turf algae and, thus, reduced algal cover. This top-down control mechanism also aligns with early findings that nitrogen limits algal community growth, but standing crop is determined by grazing losses [35], as well as with long-established theoretical expectations that subsidies support higher consumer populations, thus enabling them to further suppress resources [8]. Importantly, for natural nutrient subsidies to stimulate this top-down control and thereby reduce algal turf cover, there likely needs to be relatively intact herbivore communities. Indeed, this study was conducted across sites with minimal fishing pressure, which likely contributed to high herbivore biomass and productivity, and, thus, high rates of herbivory. While the impacts of seabird-derived nutrients in areas with few herbivores is unknown, in the Caribbean natural fish-derived nutrients can reinforce macroalgal dominance where grazing pressure is low [36]. In terms of conservation, these findings add to the body of evidence that herbivores are key to preventing coral to algae regime shifts on coral reefs (e.g., [37]), and additionally suggest that restoring ecosystem connectivity, combined with effective fisheries management, is another route to help achieve this goal.

The observed de-coupling of primary producer cover and productivity, with only productivity influencing higher trophic levels, emphasizes the need to examine both static and dynamic metrics when quantifying ecosystem structure and function. Indeed, standing crop and productivity represent distinct properties of ecosystems (e.g., [19,21,27]), with herbivorous fish biomass often correlating with turf productivity, but not turf biomass [38,39]. Despite these important differences, there is continued misapplication of “productivity” to standing crop or percent cover, likely, in part, because typical monitoring methods focus on such static metrics [40]. However, recent advances enable better estimations of productivity and other fluxes [3], and here we emphasize using dynamic measures to reveal the flow and effects of allochthonous nutrients on coral reefs. Similarly, seabird nutrient inputs have previously been shown to have no effect on coral cover until after a major climate disturbance [41], when they fueled faster recovery by increasing coral growth rates [42]. Thus, it appears that a rate-based approach, enabling the quantification of hidden pathways and processes, can reveal effects that cannot be detected with standard monitoring methods.

Here, we focus on the effects of seabird-provided subsidies on coral reefs by using δ15N as a tracer of seabird nutrients, and in doing so uncover the causal link from seabird-derived nutrients in turf algae to faster turf growth. In addition to seabirds, other consumers also affect nutrient dynamics on coral reefs and, in turn, can enhance primary productivity [43]. For example, nutrients provided by grunts (Haemulidae) and damselfishes (Pomacentridae) can boost growth rates of algae and coral, and alter benthic composition [44–48]. However, these effects tend to be localized, with changes occurring only within the fishes’ shelter sites or territories. Moreover, many coral reef fishes both feed and release their waste on the reef, and thus while important to recycling, distributing, and concentrating nutrients, are not bringing in new nutrients. Planktivores, on the other hand, are more analogous to seabirds in that they can provide an energetic and nutrient pathway from pelagic systems to coral reefs, and can boost fish productivity [49–51]. Planktivores are unlikely to be a major driver of primary productivity in this system, however, because they had low proportional biomass at our study sites (<10% of total fish biomass), which aligns with a global analysis in which Seychelles reefs had low predicted planktivore productivity [51]. Future work could aim to disentangle the relative influence of different nutrient subsidy sources on ecosystem functioning, including comparing seabirds and other consumers on reefs where both are abundant.

Beyond coral reefs, our results highlight some potential similarities, and differences, in the effects of seabird-provided nutrient subsidies on terrestrial versus marine systems. While seabird nutrients typically stimulate increases in biomass of both primary producers and consumers on islands [9,11,12,52–55], we only observed increased biomass of consumers. This difference is likely due to the strong feedback between herbivorous fish and turf algae as described above, and matches expectations that top-down control of primary producers is more important in aquatic compared to terrestrial systems [56]. However, nutrient subsidies exert strong bottom-up effects in both ecosystems, likely because most primary producers, regardless of aquatic or terrestrial habitat, experience remarkably similar limitation of nitrogen and phosphorous [57–59], in which seabird guano is rich [60–62].

Given that cross-ecosystem nutrient flows have pervasive effects in recipient ecosystems, and that invasive species including rats alter these pathways [63], removing invasive species and restoring natural nutrient subsidies should benefit multiple ecosystems. Here, seabirds influenced coral reef ecosystem functioning across multiple trophic levels. By providing a mechanistic understanding for the role of seabird-derived nutrients on coral reefs, we can now better predict the benefits of restoring cross-ecosystem nutrient subsidies.

## Materials and methods

### Study sites

This study was conducted across four granitic islands of the inner Seychelles (S1 Fig). We selected islands that encompassed a gradient of seabird biomass due to differences in rat invasion status—Aride Island and Cousine Island never had rats, Fregate Island had rats eradicated in 2001, and Félicité Island still has rats. All islands have minimal human populations and fishing pressure (S1 Table). We established one marine study site per island, except on Fregate Island where the majority of the seabirds breed on the northeast side of the island, so we chose one site on each side of the ridgeline to represent these two distinct catchments (“Fregate NE” and “Fregate SW,” S1 Fig). We opted to use this regression-style design, rather than replicating sites within islands/catchments, to maximize the range of seabird populations across our study sites and, thus, increase our ability to estimate seabird nutrient effects [64]. Study sites were all on the reef slope, 80–200 m from shore and at depths ranging from 3.9 to 5.3 m (S1 Table).

We conducted the study in November 2022, a period of extremely calm winds, which is typical during this transitional month between the SE and NW monsoon seasons (mean wind speed 2.7 m/s; S3 Fig). Although short-term exposure was, therefore, similar across our study sites, we also calculated a long-term measure of wave exposure due to its potential effects on algal turf and herbivore dynamics [65]. Hourly wind speed and direction data from 2012 to 2022 were obtained from the Seychelles Meteorological Authority. Long-term wave exposure was calculated as a function of the wind speed, wind direction, and fetch (distance of open sea over which the wind can blow to generate waves) using the methods in [66,67]. Fetch for each study site was calculated in 32 compass directions (angular width of 11.25°) using the R package *waver* *[*68*]*. We used a 10-year average of hourly wave exposure to characterize overall site exposure while accounting for strong and sporadic winds [69]. Wave exposure ranged from 140 to 483 J m^−3^ among study sites (S1 Table).

### Seabird censuses and seabird nutrient input

Seven seabird species breed across the five study islands, and censuses of breeding seabirds were conducted on each island as part of regular monitoring programs (S2 Table). We estimated seabird populations using the survey conducted during the main breeding season immediately before the time of marine data collection, which was June–August 2022 for lesser noddies (*Anous tenuirostris*), brown noddies (*Anous stolidus*), white-tailed tropicbirds (*Phaethon lepturus*), white/fairy terns (*Gygis alba*), and sooty terns (*Onychoprion fuscatus*), and November 2021–February 2022 for wedge-tailed shearwaters (*Ardenna pacifica*) and tropical shearwaters (*Puffinus bailloni*). Breeding pairs and nests for the majority of seabird species were counted using 300 m^2^ circular plots. Exceptions to this were shearwaters and sooty terns on Aride Island and lesser noddies on Fregate Island which were censused by conducting plots within known mapped territories. Breeding pair density was converted to seabird breeding biomass (kg/ha/year) by using species-specific mean adult mass to convert density to biomass [70] and then scaling by the proportion of the year each species breeds on these islands. This biomass metric is more relevant than density for approximating seabird nutrient inputs to island and nearshore ecosystems [26,71]. To better encompass the nutrient inputs most likely to flow to our marine study sites, we divided each island area by the ridgeline, and only used seabird biomass in the catchment nearest to each site.

We used the ratio of the nitrogen isotope N15:N14 relative to standard atmospheric nitrogen (expressed as δ15N) as an indicator of seabird-derived nutrients in primary producers. Samples, including coastal plants and marine algae, collected near sites with large seabird populations elsewhere in the Indo-Pacific are consistently higher in δ15N compared to nearby reference sites where seabirds are few or absent, reflecting the high δ15N values in seabird guano [26,71–74]. Thus, enhanced δ15N in primary producers indicates increased exposure to and assimilation of seabird-derived nutrients, and can also indicate increased resource quality [75]. We, therefore, collected 10 new growth leaves of the coastal plant *Scaevola taccada,* picked from plants located along the beach adjacent to each marine study site for stable isotope analysis as an indicator of seabird-derived nutrients flowing to these sites. All leaves were dried at 60 °C for 48 h, and analyzed at The University of Texas at Austin—Marine Science Institute, Core Isotope Facility in Port Aransas, Texas using a Thermo Fisher Scientific Flash EA-Isolink CNSOH elemental analyzer connected to a Thermo Fisher Scientific Delta V Plus isotope-ratio-mass spectrometer. A two-point calibration of *δ*15N to AIR was achieved using USGS-41a (+36.55‰, + 47.55‰), and internal laboratory standards of casein protein and glycine were used to evaluate precision and accuracy (standard deviation < 0.2‰).

### Primary producer measurements

We focused on algal turfs, which are multispecies assemblages of filamentous algae < 2 cm tall (also called “epithelic algal communities” or “turf algae”), because they are a main component of coral reef benthic cover and productivity, and they constitute a major trophic pathway from benthic production to fish [27,28,76].

Following [77–80], we measured primary productivity as the change in algal turf height within herbivore exclusion cages over a period of 4–7 days. Briefly, we selected areas of hard substrate on the reef slope (3.9–5.3 m deep) that were covered in algal turf while avoiding the territories of farming damselfishes and sediment-laden areas, as these can strongly influence turf dynamics [47,80,81]. We conducted 10 replicates per site, and for each replicate we measured turf height at 10 haphazardly chosen points within a 14 × 14 cm area using the depth probe of vernier calipers. Because the depth probe of vernier calipers is the same length as the distance between the caliper tips, we pressed the caliper tips into saltwater-resistant adhesive putty (Blu-tack) between each height measurement to quickly record height underwater and enable more accurate post-dive measurements using digital calipers. Immediately after each dive, indentations were measured using digital calipers and averaged to obtain one initial turf height measurement (mm) for each replicate. Over each area of substrate, we placed a cage measuring 14 cm × 14 cm × 10 cm (length × width × height) made of PVC-plastic with 1.2 cm openings (S6 Fig). Each cage had a 5-cm fringe around all sides through which nails were hammered into the substrate. Similar cages have successfully been used to exclude herbivores and measure turf growth in previous studies [38,39,77–80]. Cage controls were not used because caging artifacts are expected to be minimal over the short duration of these experiments [39,80], and based on extensive previous work testing for caging artifacts using similar cages [77,82,83]. In addition, the same cages were applied to all replicates, and, thus, turf growth rates quantified here are comparable to one another (i.e., relative growth) but may be lower than natural growth rates in the absence of cages (e.g., due to shading, reduced water flow, increased sediment deposition, and/or the asymptotic slowing of growth as turfs lengthen) [80,82]. At the conclusion of the experiment, we removed the cages and remeasured turf height at 10 haphazardly selected points as above. Primary productivity (mm/day) per replicate cage was calculated as the change in mean turf height divided by the number of days between surveys. These methods, with similar levels of replication, have been widely used to measure turf productivity on reefs [77–80,84]. During the final measurements, we additionally used a dive knife to remove a sample of algal turf communities from inside each cage and collected these in small plastic bags to analyze δ15N values as for leaves above. When we returned to remeasure, two cages were missing, and two cages appeared barer within the cage than immediately outside the cage, which was in stark contrast to the majority of replicates where there was clear turf growth within the cages (S6 Fig). These two replicates also had final height measurements that were shorter than initial measurements, suggesting that herbivores accessed cages during the experiment, possibly through gaps under the cages. These four replicates were excluded from all analyses, resulting in 46 total replicates (*n* = 8 at Fregate SW, 9 at Cousine, 9 at Félicité, 10 at Fregate NE, and 10 at Aride).

Initial turf heights were similar at Felicite, Fregate NE, Cousine, and Aride, while Fregate SW had taller turfs (mean initial height = 1.37, 1.92, 1.89, 1.42, and 3.48 mm, respectively). Because initial turf height can affect turf growth, with shorter turfs growing faster than longer turfs [80], we also calculated proportional turf growth for each cage (change in turf height per day/initial turf height) and compared models with this as a response to those with absolute growth as a response. The results were nearly identical (see Results), so we proceeded with absolute growth to match previous studies and because it is more relevant to herbivores.

Algal turf cover was quantified along four replicate 30-m transects at each site, conducted in the same area as the turf productivity cages on the reef slope. One observer recorded benthic cover every 50 cm using the point-intercept method, which we used to calculate % turf cover. Turf algae was the dominant primary producer across all sites, and macroalgae was extremely rare (overall mean macroalgae cover = <0.5%, mean crustose coralline algae [CCA] cover = 5.3%, mean turf cover = 13.5%). Coral cover ranged from 10% to 55% among sites. We also estimated turf standing crop by multiplying % turf cover along each transect by initial turf algal height from the productivity cages. Because % cover and standing crop were highly correlated, we proceeded with % cover for analyses. Structural complexity, which is often negatively associated with algal cover and positively associated with herbivore biomass and productivity [85], was also visually estimated along each transect using a standard scale ranging from 0 (no relief) to 5 (exceptionally complex relief) [86,87].

### Primary consumer measurements

Herbivorous fishes are the main consumers of benthic algae on coral reefs, and were surveyed along the same transects as algal turf cover. The same experienced observer (NAJG) recorded the species and total length (estimated to the nearest cm) of all diurnal, non-cryptic reef fishes along each transect. Larger, more active fish were counted within a 5-m wide belt while laying down the transect during a first pass, and site-attached damselfishes (Pomacentridae) within a 2-m wide belt transect during a second pass. Biomass of each fish was calculated using published species-specific Bayesian length–weight relationships from Fishbase [88], and total herbivorous fish standing biomass was summed along each transect (kg/ha).

Recent modeling advances enable reef fish productivity to be calculated at both individual and assemblage levels by combining underwater survey data with predicted growth and size-based mortality rates [3]. Growth trajectories for each fish species were predicted using a trait-based approach and a mean sea surface temperature of 28 °C using the *rfishprod* R package [20,89]. Each individual herbivorous fish from the surveys was placed within its growth trajectory to estimate somatic growth (g/day). Productivity was calculated as the sum of biomass gained along each transect (kg/ha/day).

We additionally divided herbivores into finer feeding groups to test the hypothesis that herbivores that specifically feed on turf algae are expected to show stronger positive relationships with turf cover and productivity than other herbivores. We used classifications based on “how” and “what” they eat [1]—croppers (turf algae), brushers (detritus and sediments within the epilithic algal matrix), sediment suckers (detritus and sediments from soft or mixed substrata), scrapers (microscopic cyanobacteria on or within reef substrate), excavators (microscopic cyanobacteria on or deep within reef substrate), farmers (turf algae within defended territories), and browsers (macroalgae) (S3 Table). We also classified herbivores into their functional role, divided by whether or not they remove turf algae based on their feeding mode and feeding habitat, to test the hypothesis that turf cover will be more negatively associated with herbivores that function to remove turf algae (S3 Table).

### Ethics statement

Fieldwork was conducted with approval from the Seychelles Bureau of Standards (Permit A0157).

### Statistical analysis

We used a structural causal modeling (SCM) approach, which enables causal inference from observational data, and has been used extensively in other fields but only recently applied in ecology [90]. We first created a DAG to represent causal relationships within our system (S1 Fig). We were primarily interested in determining the causal effects of seabird nutrients on turf algal nutrients, and, in turn, on turf cover and growth, and herbivore biomass and growth (Fig 1). We controlled for additional variables that are known to influence these dynamics in our study design, including reef zone, distance to shore, depth, human nutrient pollution, and fishing pressure by selecting sites on the reef slope at similar distances to shore (80–200 m) and depths (3.9–5.3 m), with minimal nearby human populations and fishing pressure (S1 Table). Additional variables that are important to understanding the system, as well as common causes of pairs of variables, were included in our DAG. These included island size, invasive rat status, turf height, wave energy, coral cover, structural complexity, and predator biomass, which varied among sites (S1 Fig). In addition to the DAG presented here, we considered a DAG which included connections between productivity and biomass within trophic levels. However, we decided to leave these out because of evidence for de-coupling of turf productivity and cover and fish biomass and productivity [19–21].

We accounted for potential feedbacks between turf algae and herbivores in several ways. First, we included a temporal element in our DAG, which enables the inclusion of bidirectional relationships or feedback without violating the need for acyclic models [90]. Specifically, we initially assumed that top-down effects of prior herbivory influenced current turf characteristics, which, in turn, would have bottom-up effects on current herbivore communities. The focus on bottom-up effects of turf on herbivores during the current time point (time of sampling) was chosen because the main focus of this study was to determine the effect of cross-ecosystem nutrient subsidies on primary producers and consumers. Furthermore, there is previous evidence for effects of seabird nutrients on algal nutrients and herbivorous reef fishes and for bottom-up effects of cross-ecosystem nutrient subsidies more generally, suggesting this direction of causality is reasonable *a priori* [8,9,11,25,26,75].

To further test the robustness of our findings, after testing all of our hypotheses we then made a new DAG based on our results and proposed balance between bottom-up and top-down effects. Specifically, we suggest that by increasing primary productivity, seabird nutrients increased herbivore biomass and productivity, which, in turn, exert top-down control on turf algal cover. This alternative DAG was reasonable based on the study design whereby primary productivity was measured in cages and, thus, was unlikely to be influenced by current herbivory, while turf algal cover was surveyed along transects (outside of cages) so could be rapidly influenced by current herbivores.

After creating our DAGs, we checked the validity of the assumptions and determined adjustment sets required to close biasing paths and test individual causal pathways using the package *dagitty* [91] in R [92] (S4 and S5 Tables). Specifically, we tested the following causal hypotheses: Seabirds increase nutrients of coastal leaves (H1) and algal turfs (H2). Higher turf nutrients lead to faster turf growth (H3) and higher turf cover (H4). Faster turf growth leads to higher herbivore biomass (H5) and productivity (H6). Higher turf cover leads to higher herbivore biomass (H7) and productivity (H8). Higher turf nutrients lead to higher herbivore biomass (H9) and productivity (H10). For hypotheses H9 and H10, turf nutrients could affect herbivores either directly (due to increased food quality) and/or indirectly via their effects on turf growth (i.e., food replenishment) and/or turf cover (i.e., food quantity). Therefore, we compared the direct effect (food quality only) to the total effect (food quality + food quantity + food replenishment) of turf nutrients on herbivore biomass and productivity to elucidate the pathway(s) by which seabird nutrients in algal turfs influence consumers. We then used the alternative DAG to test the same causal hypotheses, with the exception that H7 and H8 were reversed to test the hypotheses that herbivore productivity and biomass have negative effects on turf cover via top-down controls (S1B Fig and S5 Table).

We used separate Bayesian models to test each hypothesized causal relationship, while accounting for island as a group-level effect [93] (S4 and S5 Tables). All responses, with the exception of turf productivity, were log-transformed, after which all responses followed normal (Gaussian) distributions. We used weakly informative priors, and ran models for four chains with 3,000 iterations and a warm-up of 1,000 iterations per chain, using the *brms* package in R, implemented in Stan [94,95]. Model convergence and fit were checked using traceplots, posterior predictive plots, and the Gelman–Ruban convergence diagnostic (R-hat) [93]. All models had high convergence (R-hat = 1.00) and fit well, but the limited replication across sites with similar seabird populations in our study design may have contributed to wide uncertainty intervals in some analyses. For each hypothesized causal pathway, we calculated the estimated effect size and 95% HPDI using the *tidybayes* package, and determined the posterior probability under the hypothesis (PP) using the *brms* hypothesis() function [94–96].

Finally, we used several correlative analyses to complement our DAG-based approach and further examine the robustness of our findings. We used a correlation analysis to examine the relationships among our variables of interest without assuming causation or directionality. We then used multivariate analyses (nonmetric multidimensional scaling, NMDS) to visualize functional communities of herbivorous fishes in species space using the R package *vegan* [97]. By overlaying environmental variables expected to influence herbivores that were not controlled for in our study design (exposure, structural complexity, predator biomass), along with our variables of interest (turf nutrients, turf productivity, turf cover), we examined which variables were most correlated with which functional groups. We also ran an NMDS of benthic communities to examine how turf cover correlated with variables expected to influence turf cover that were not controlled for in our study design (exposure, structural complexity), along with our variables of interest (biomass and productivity of herbivores, broken down by whether they function to remove turf algae).

## Supporting information

S1 Table Characteristics of the five study sites. (PDF)

S2 Table Seabird biomass by species across the five study sites. All values are breeding biomass scaled by the proportion of year each species breeds on the islands (kg/ha/year).
(PDF)

S3 Table Fine feeding groups for herbivorous fish species observed in this study. Fishes were classified by how they feed, what they feed on, and whether they function to remove turf algae, following Bellwood and colleagues (2019) and Tebbett and colleagues (2022).
(PDF)

S4 Table Details of statistical models used to test causal pathways in the original DAG (S1A Fig). Separate models were used to test each causal pathway, including both the direct causal path and the total path (which includes all possible direct + indirect pathways). Adjustment sets show additional co-variates included in the model to close biasing paths. Estimated effect sizes and 95% highest posterior density intervals are the untransformed results from Bayesian models, along with the hypothesized direction of each effect (“expected effect”) and the posterior probability of the expected effect.
(PDF)

S5 Table Details of statistical models used to test causal pathways in the alternative directed acyclic graph (DAG) (S1B Fig). Separate models were used to test each causal pathway, including both the direct causal path and the total path (which includes all possible direct + indirect pathways). Adjustment sets show additional co-variates included in the model to close biasing paths. Estimated effect sizes and 95% highest posterior density intervals are the untransformed results from Bayesian models, along with the hypothesized direction of each effect (“expected effect”) and the posterior probability of the expected effect. Comparison notes compare the results from these models to those presented in S4 Table based on the original DAG (S1A Fig).
(PDF)

S1 Fig Directed acyclic graphs (DAGs) of causal relationships between seabird nutrients, turf algae, and herbivores. Thick arrows represent causal hypotheses of interest, and thin arrows represent all other hypothesized causal relationships. **(A)** Original DAG, assuming only bottom-up effects. **(B)** Alternative DAG, assuming a combination of bottom-up and top-down effects. Gray boxes with dashed borders indicate unmeasured variables. Prior herbivory and prior turf help incorporate bidirectional relationships into our DAGs, emphasizing the hypothesized directional effects at the time of sampling. Both are assumed to be affected by exposure, because this is a 10-year average, so stable throughout the study. In addition to the DAGs presented here, we also built DAGs that included the variables we controlled for in our study design (e.g., depth, reef zone, distance to shore, fishing pressure). The inclusion of these variables does not change the statistical models used (S4 and S5 Tables) but make it more difficult to visualize the DAGs, so we did not display them here.
(PDF)

S2 Fig Map of study sites, showing location on world map (top), and zoomed into the inner Seychelles (bottom). Maps were created in R with associated packages *ggplot2*, *sf*, and *ggspatial*. Seychelles shape file was obtained from https://data.humdata.org/dataset/cod-ab-syc under their CC-BY-IGO license.
(PDF)

S3 Fig Rose diagram of hourly wind direction and speed by month for 2022. The study period (November 2022) is indicated by a thick orange border around the panel. The data underlying this figure are from the Seychelles International Airport provided by the Seychelles Meteorological Authority and can be found in https://doi.org/10.5281/zenodo.15485420.
(PDF)

S4 Fig Correlations among algal turf and herbivorous fish metrics. Turf nutrients are the amount of seabird-derived nutrients in turf algae (measured as δ^15^N), turf productivity is growth (mm/day, measured in herbivore exclusion cages), turf cover is proportional cover along benthic transects (log-transformed), herbivore productivity is calculated from fish transects (log kg/ha/day), and herbivore biomass is from fish transects (log kg/ha). All metrics are site-level means. The data underlying this figure can be found in https://doi.org/10.5281/zenodo.15485420.
(PDF)

S5 Fig Non-metric multidimensional scaling (NMDS) plots of herbivorous fish (A-B) and benthic (C) communities. **(A, B)** Herbivorous fish were grouped into functional groups based on how they feed, with community biomass in (A) and community productivity in (B). Colored circles represent transects, with the distance between points approximating community dissimilarity (i.e., points that are closer together have more similar communities, points that are farther apart have more different communities). Shaded areas represent minimum convex hull polygons for each site. Black triangles and text indicate the position of herbivore functional groups (A, B) and benthic groups (C). Gray arrows and text indicate the strength and direction of effects of environmental correlates. The data underlying this figure can be found in https://doi.org/10.5281/zenodo.15485420. (A) Turf productivity, turf nutrients, and structure were the environmental variables most associated with herbivorous fish community biomass (*r*^2^ = 0.52, 0.31, 0.26), and all showed positive associations with NMDS1. Turf-feeding croppers were the functional group most positively correlated with NMDS1 (cor = 0.76), and, thus, with these environmental variables, with macroalgal browsers and detritivorous brushers also showing positive correlations with NMDS1 (cor = 0.51, 0.53), while farming damselfishes showed a strong negative correlation with NMDS 1 (cor = −0.64). (B) Structure, turf productivity, and turf nutrients also showed strong associations with herbivorous fish community productivity (*r*^2^ = 0.47, 0.36, 0.26). Cropper productivity was the most associated with NMDS1 (cor = 0.86), with browsers also showing a strong positive association (cor = 0.72). Scrapers and farmers had the strongest negative associations with NMDS 1 (cor = −0.59, −0.54). **(C)** Exposure, turf nutrients, structure, and biomass of turf-removing herbivores were the environmental variables most associated with benthic communities (*r*^2^ = 0.69, 0.54, 0.34, 0.26), and all showed positive associations with NMDS1. Turf algae and sand/rubble were the benthic groups most negatively correlated with NMDS1 (cor = −0.36, −0.74, respectively), while hard coral was most positively correlated with NMDS1 (cor = 0.96).
(PDF)

S6 Fig Photographs of herbivore exclusion cages used for measurement of algal turf productivity. **(a)** Example herbivore exclusion cage one day after installation. Each cage was 14 × 14 × 10 cm with 1.2 cm openings and a 5-cm fringe around all sides through which nails were hammered into the substrate. **(b)** Example herbivore exclusion cage upon removal after 4–7 days. Turf algae grows quickly in the absence of herbivores, resulting in clear differences between the areas within the cage and immediately adjacent to the cage by the end of the experiment.
(PDF)

## Abbreviations

DAG: directed acyclic graph
HPDI: highest posterior density interval
NMDS: nonmetric multidimensional scaling
PP: posterior probability of expected effect
SCM: structural causal modeling
