# Supplementary material for: Nutrient connectivity via seabirds enhances dynamic measures of coral reef ecosystem function
Source: PLoS Biol. 2025 Jul 8;23(7):e3003222. doi: 10.1371/journal.pbio.3003222 (PMC12237027; doi:10.1371/journal.pbio.3003222)
Supplement: S1 Table — (PDF) [file pbio.3003222.s001.pdf]

***S1 Table.* Characteristics of the five study sites.**

| | Aride Island | Cousine Island | Félicité Island | Fregate Island SW | Fregate Island NE |
|---|---|---|---|---|---|
| **Island size (ha)** | 71 | 26 | 268 | 206 | 206 |
| **Rat status** | never introduced | never introduced | present | eradicated | eradicated |
| **Human population** | minimal (conservation station) | minimal (luxury resort) | minimal (luxury resort) | minimal (luxury resort) | minimal (luxury resort) |
| **Fishing pressure** | none | minimal | minimal | minimal | minimal |
| **Reef zone** | reef slope | reef slope | reef slope | reef slope | reef slope |
| **Distance to shore (m)** | 120 | 130 | 80 | 180 | 200 |
| **Depth (m)** | 5.3 | 4.4 | 3.9 | 5.0 | 4.9 |
| **Wave energy (J $m^{-3}$, 10-year average)** | 140 | 483 | 168 | 470 | 379 |
