# Supplementary material for: Nutrient connectivity via seabirds enhances dynamic measures of coral reef ecosystem function
Source: PLoS Biol. 2025 Jul 8;23(7):e3003222. doi: 10.1371/journal.pbio.3003222 (PMC12237027; doi:10.1371/journal.pbio.3003222)
Supplement: S2 Table — All values are breeding biomass scaled by the proportion of year each species breeds on the islands (kg/ha/year). (PDF) [file pbio.3003222.s002.pdf]

S2 Table. **Seabird biomass by species across the five study sites.** All values are breeding biomass scaled by the proportion of year each species breeds on the islands (kg/ha/year).

	**Aride Island**	**Cousine Island**	**Félicité Island**	**Fregate Island SW**	**Fregate Island NE**
**Brown noddy (*Anous stolidus*)**	3.10	2.25	0.00	0.00	0.00
**Lesser noddy (*Anous tenuirostris*)**	61.50	107.31	0.00	2.88	43.90
**Sooty tern (*Onychoprion fuscatus*)**	6.59	0.00	0.00	0.00	0.00
**White/fairy tern (*Gygis alba*)**	3.00	3.34	0.00	1.02	2.36
**Wedge-tailed shearwater (*Ardenna pacifica*)**	31.69	32.80	0.00	0.00	0.00
**Tropical shearwater (*Puffinus bailloni*)**	34.64	0.00	0.00	0.00	0.00
**White-tailed tropicbird (*Phaethon lepturus*)**	5.16	10.50	0.00	0.00	0.00
**Total**	145.69	156.21	0.00	3.90	46.25
