# Supplementary material for: Nutrient connectivity via seabirds enhances dynamic measures of coral reef ecosystem function
Source: PLoS Biol. 2025 Jul 8;23(7):e3003222. doi: 10.1371/journal.pbio.3003222 (PMC12237027; doi:10.1371/journal.pbio.3003222)
Supplement: S3 Table — Fishes were classified by how they feed, what they feed on, and whether they function to remove turf algae, following Bellwood and colleagues (2019) and Tebbett and colleagues (2022). (PDF) [file pbio.3003222.s003.pdf]

S3 Table. **Fine feeding groups for herbivorous fish species observed in this study.** Fishes were classified by how they feed, what they feed on, and whether they function to remove turf algae, following Bellwood et al. (2019) and Tebbett et al. (2022).

**Family**	**Species**	**How?**	**What?**	**Turf removal function?**
Acanthuridae	*Acanthurus leucosternon*	Cropper	Turf algae	Yes
Acanthuridae	*Acanthurus lineatus*	Cropper	Turf algae	Yes
Acanthuridae	*Acanthurus nigricauda*	Sediment sucker	Detritus and sediments	Yes
Acanthuridae	*Acanthurus nigrofuscus*	Cropper	Turf algae	Yes
Acanthuridae	*Acanthurus tennentii*	Sediment sucker	Detritus and sediments	Yes
Acanthuridae	*Acanthurus triostegus*	Cropper	Turf algae	Yes
Acanthuridae	*Acanthurus xanthopteus*	Sediment sucker	Detritus and sediments	Yes
Acanthuridae	*Ctenochaetus binotatus*	Brusher	Detritus and sediments	No
Acanthuridae	*Ctenochaetus striatus*	Brusher	Detritus and sediments	No
Acanthuridae	*Ctenochaetus truncatus*	Brusher	Detritus and sediments	No
Acanthuridae	*Naso brachycentron*	Browser	Macroalgae	No
Acanthuridae	*Naso elegans*	Browser	Macroalgae	No
Acanthuridae	*Zebrasoma desjardinii*	Cropper	Turf algae	Yes
Acanthuridae	*Zebrasoma scopas*	Cropper	Turf algae	Yes
Kyphosidae	*Kyphosus cinerascens*	Browser	Macroalgae	No
Labridae: Scarini	*Calotomus carolinus*	Browser	Macroalgae	No
Labridae: Scarini	*Chlorurus atrilunula*	Excavator	Microscopic cyanobacteria	Yes
Labridae: Scarini	*Chlorurus sordidus*	Excavator	Microscopic cyanobacteria	Yes
Labridae: Scarini	*Chlorurus strongylocephalus*	Excavator	Microscopic cyanobacteria	Yes
Labridae: Scarini	*Scarus caudofasciatus*	Scraper	Microscopic cyanobacteria	Yes
Labridae: Scarini	*Scarus falcipinnis*	Scraper	Microscopic cyanobacteria	Yes
Labridae: Scarini	*Scarus frenatus*	Scraper	Microscopic cyanobacteria	Yes
Labridae: Scarini	*Scarus ghobban*	Scraper	Microscopic cyanobacteria	Yes
Labridae: Scarini	*Scarus niger*	Scraper	Microscopic cyanobacteria	Yes
Labridae: Scarini	*Scarus prasiognathus*	Scraper	Microscopic cyanobacteria	Yes
Labridae: Scarini	*Scarus psittacus*	Scraper	Microscopic cyanobacteria	Yes
Labridae: Scarini	*Scarus rubroviolaceus*	Scraper	Microscopic cyanobacteria	Yes
Labridae: Scarini	*Scarus tricolor*	Scraper	Microscopic cyanobacteria	Yes
Labridae: Scarini	*Scarus viridifucatus*	Scraper	Microscopic cyanobacteria	Yes
Pomacentridae	*Stegastes lacrymatus*	Farmer	Turf algae (within defended territories)	No
Pomacentridae	*Stegastes nigricans*	Farmer	Turf algae (within defended territories)	No
Siganidae	*Siganus argenteus*	Browser	Macroalgae	No
Siganidae	*Siganus stellatus*	Cropper	Turf algae	Yes
Siganidae	*Siganus sutor*	Browser	Macroalgae	No
