# Supplementary material for: Nutrient connectivity via seabirds enhances dynamic measures of coral reef ecosystem function
Source: PLoS Biol. 2025 Jul 8;23(7):e3003222. doi: 10.1371/journal.pbio.3003222 (PMC12237027; doi:10.1371/journal.pbio.3003222)
Supplement: S4 Table — Separate models were used to test each causal pathway, including both the direct causal path and the total path (which includes all possible direct + indirect pathways). Adjustment sets show additional co-variates included in the model to close biasing paths. Estimated effect sizes and 95% highest posterior density intervals are the untransformed results from Bayesian models, along with the hypothesized direction of each effect (“expected effect”) and the posterior probability of the expected effect. (PDF) [file pbio.3003222.s004.pdf]

S4 Table. **Details of statistical models used to test causal pathways in the original DAG (S1A Fig).** Separate models were used to test each causal pathway, including both the direct causal path and the total path (which includes all possible direct + indirect pathways). Adjustment sets show additional co-variates included in the model to close biasing paths. Estimated effect sizes and 95% highest posterior density intervals (HPDIs) are the untransformed results from Bayesian models, along with the hypothesized direction of each effect ('expected effect') and the posterior probability of the expected effect.

Hypothesis	Causal pathway	Pathway type	Adjustment set	Effect size (95% HPDI)	Expected effect	Posterior probability of expected effect
H1	seabirds -> leaf nutrients	direct & total	island size	0.16 (0.10,0.22)	+	>0.99
H2	seabirds -> turf nutrients	direct & total	island size	0.08 (-0.02,0.18)	+	0.94
H3	turf nutrients -> turf productivity	direct	exposure, turf height	0.04 (-0.01,0.10)	+	0.94
H3	turf nutrients -> turf productivity	total	exposure	0.05 (0.00,0.11)	+	0.97
H4	turf nutrients -> turf cover	direct & total	exposure	-0.40 (-1.16,0.45)	+	0.15
H5	turf productivity -> herbivore productivity	direct & total	exposure, structure, turf nutrients, turf cover	0.45 (-2.93,3.20)	+	0.71
H6	turf productivity -> herbivore biomass	direct & total	exposure, structure, turf nutrients, turf cover	0.66 (-3.76,4.79)	+	0.70
H7	turf cover -> herbivore productivity	direct & total	exposure, structure, turf nutrients, turf productivity	-0.09 (-0.34,0.18)	+	0.24
H8	turf cover -> herbivore biomass	direct & total	exposure, structure, turf nutrients, turf productivity	-0.07 (-0.35,0.19)	+	0.28
H9	turf nutrients -> herbivore productivity	direct	exposure, structure, turf cover, turf productivity	-0.20 (-1.79,1.36)	+	0.35
H9	turf nutrients -> herbivore productivity	total	exposure	0.16 (-0.53,0.87)	+	0.72
H10	turf nutrients -> herbivore biomass	direct	exposure, structure, turf cover, turf productivity	0.03 (-1.88,2.20)	+	0.52
H10	turf nutrients -> herbivore biomass	total	exposure	0.41 (-0.45,1.36)	+	0.85
