# Supplementary material for: Nutrient connectivity via seabirds enhances dynamic measures of coral reef ecosystem function
Source: PLoS Biol. 2025 Jul 8;23(7):e3003222. doi: 10.1371/journal.pbio.3003222 (PMC12237027; doi:10.1371/journal.pbio.3003222)
Supplement: S5 Table — Separate models were used to test each causal pathway, including both the direct causal path and the total path (which includes all possible direct + indirect pathways). Adjustment sets show additional co-variates included in the model to close biasing paths. Estimated effect sizes and 95% highest posterior density intervals are the untransformed results from Bayesian models, along with the hypothesized direction of each effect (“expected effect”) and the posterior probability of the expected effect. Comparison notes compare the results from these models to those presented in S4 Table based on the original DAG (S1A Fig). (PDF) [file pbio.3003222.s005.pdf]

***S5 Table.* Details of statistical models used to test causal pathways in the alternative DAG (S1B Fig).** Separate models were used to test each causal pathway, including both the direct causal path and the total path (which includes all possible direct + indirect pathways). Adjustment sets show additional co-variates included in the model to close biasing paths. Estimated effect sizes and 95% highest posterior density intervals (HPDIs) are the untransformed results from Bayesian models, along with the hypothesized direction of each effect ('expected effect') and the posterior probability of the expected effect. Comparison notes compare the results from these models to those presented in S4 Table based on the original DAG (S1A Fig).

Hypothesis	Causal pathway	Pathway type	Adjustment set	Effect size (95% HPDI)	Expected effect	Posterior probability of expected effect	Comparison notes
H1	seabirds -> leaf nutrients	direct & total	island size	0.16 (0.10,0.22)	+	>0.99	identical
H2	seabirds -> turf nutrients	direct & total	island size	0.08 (-0.02,0.18)	+	0.94	identical
H3	turf nutrients -> turf productivity	direct	exposure, turf height	0.04 (-0.01,0.10)	+	0.94	identical
H3	turf nutrients -> turf productivity	total	exposure	0.05 (0.00,0.11)	+	0.97	identical
H4	turf nutrients -> turf cover	direct	n/a	n/a	+	n/a	no longer possible to get direct effect due to top-down effects of herbivory (unobserved)
H4	turf nutrients -> turf cover	total	exposure	-0.40 (-1.16,0.45)	-	0.85	now hypothesized negative total effect if turf nutrients stimulate top-down control, but similar adjustment set and estimates
H5	turf productivity -> herbivore productivity	direct & total	exposure, turf nutrients	0.44 (-2.68,3.13)	+	0.73	different adjustment set, but similar estimates
H6	turf productivity -> herbivore biomass	direct & total	exposure, turf nutrients	0.62 (-3.54,5.05)	+	0.70	different adjustment set, but similar estimates
H7b	herbivore productivity -> turf cover	total	exposure, structure, turf nutrients, turf productivity, predators	-0.55 (-1.55,0.43)	-	0.88	new causal pathway (top-down effect)
H8b	herbivore biomass -> turf cover	total	exposure, structure, turf nutrients, turf productivity, predators	-0.49 (-1.43,0.47)	-	0.85	new causal pathway (top-down effect)
H9	turf nutrients -> herbivore productivity	direct	exposure, turf productivity	-0.13 (-1.61,1.47)	+	0.40	different adjustment set, but similar estimates
H9	turf nutrients -> herbivore productivity	total	exposure	0.16 (-0.53,0.87)	+	0.72	identical
H10	turf nutrients -> herbivore biomass	direct	exposure, turf productivity	0.12 (-1.95,2.24)	+	0.56	different adjustment set, but similar estimates
H10	turf nutrients -> herbivore biomass	total	exposure	0.41 (-0.45,1.36)	+	0.85	identical
