# Supplementary material for: Nutrient connectivity via seabirds enhances dynamic measures of coral reef ecosystem function
Source: PLoS Biol. 2025 Jul 8;23(7):e3003222. doi: 10.1371/journal.pbio.3003222 (PMC12237027; doi:10.1371/journal.pbio.3003222)
Supplement: S1 Fig — Thick arrows represent causal hypotheses of interest, and thin arrows represent all other hypothesized causal relationships. (A) Original DAG, assuming only bottom-up effects. (B) Alternative DAG, assuming a combination of bottom-up and top-down effects. Gray boxes with dashed borders indicate unmeasured variables. Prior herbivory and prior turf help incorporate bidirectional relationships into our DAGs, emphasizing the hypothesized directional effects at the time of sampling. Both are assumed to be affected by exposure, because this is a 10-year average, so stable throughout the study. In addition to the DAGs presented here, we also built DAGs that included the variables we controlled for in our study design (e.g., depth, reef zone, distance to shore, fishing pressure). The inclusion of these variables does not change the statistical models used (S4 and S5 Tables) but make it more difficult to visualize the DAGs, so we did not display them here. (PDF) [file pbio.3003222.s006.pdf]

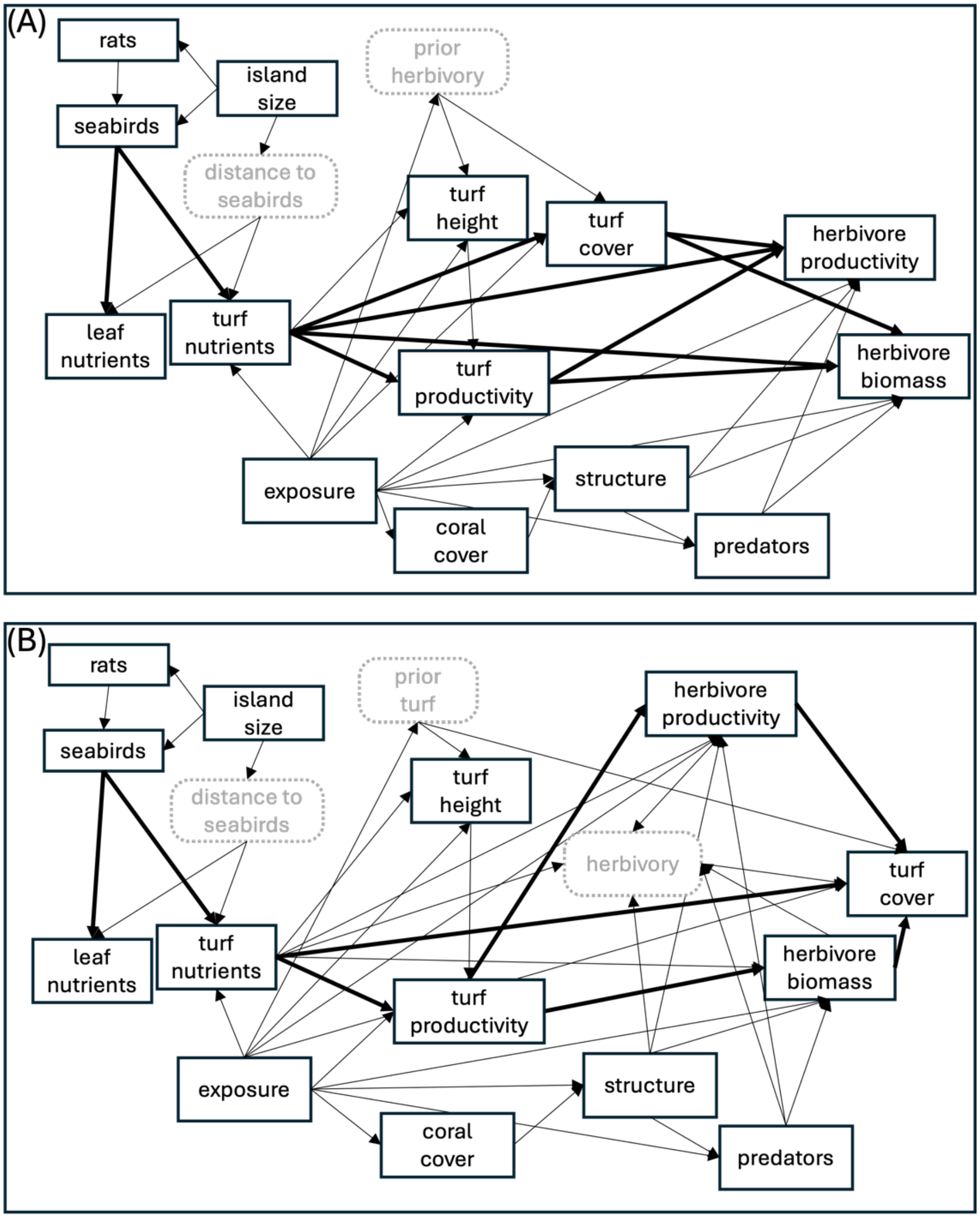

S1 Fig. **Directed acyclic graphs (DAGs) of causal relationships between seabird nutrients, turf algae, and herbivores.** Thick arrows represent causal hypotheses of interest, thin arrows represent all other hypothesized causal relationships. (A) Original DAG, assuming only bottom-up effects. (B) Alternative DAG, assuming a combination of bottom-up and top-down effects. Gray boxes with dashed borders indicate unmeasured variables. Prior herbivory and prior turf help incorporate bidirectional relationships into our DAGs, emphasizing the hypothesized directional effects at the time of sampling. Both are assumed to be affected by exposure, because this is a 10-year average, so stable throughout the study. In addition to the DAGs presented here, we also built DAGs that included the variables we controlled for in our study design (e.g., depth, reef zone, distance to shore, fishing pressure). The inclusion of these variables does not change the statistical models used (S4-S5 Tables), but does make it more difficult to visualize the DAGs, so we did not display them here.
