# Supplementary material for: Nutrient connectivity via seabirds enhances dynamic measures of coral reef ecosystem function
Source: PLoS Biol. 2025 Jul 8;23(7):e3003222. doi: 10.1371/journal.pbio.3003222 (PMC12237027; doi:10.1371/journal.pbio.3003222)
Supplement: S2 Fig — Maps were created in R with associated packages ggplot2, sf, and ggspatial. Seychelles shape file was obtained from https://data.humdata.org/dataset/cod-ab-syc under their CC-BY-IGO license. (PDF) [file pbio.3003222.s007.pdf]

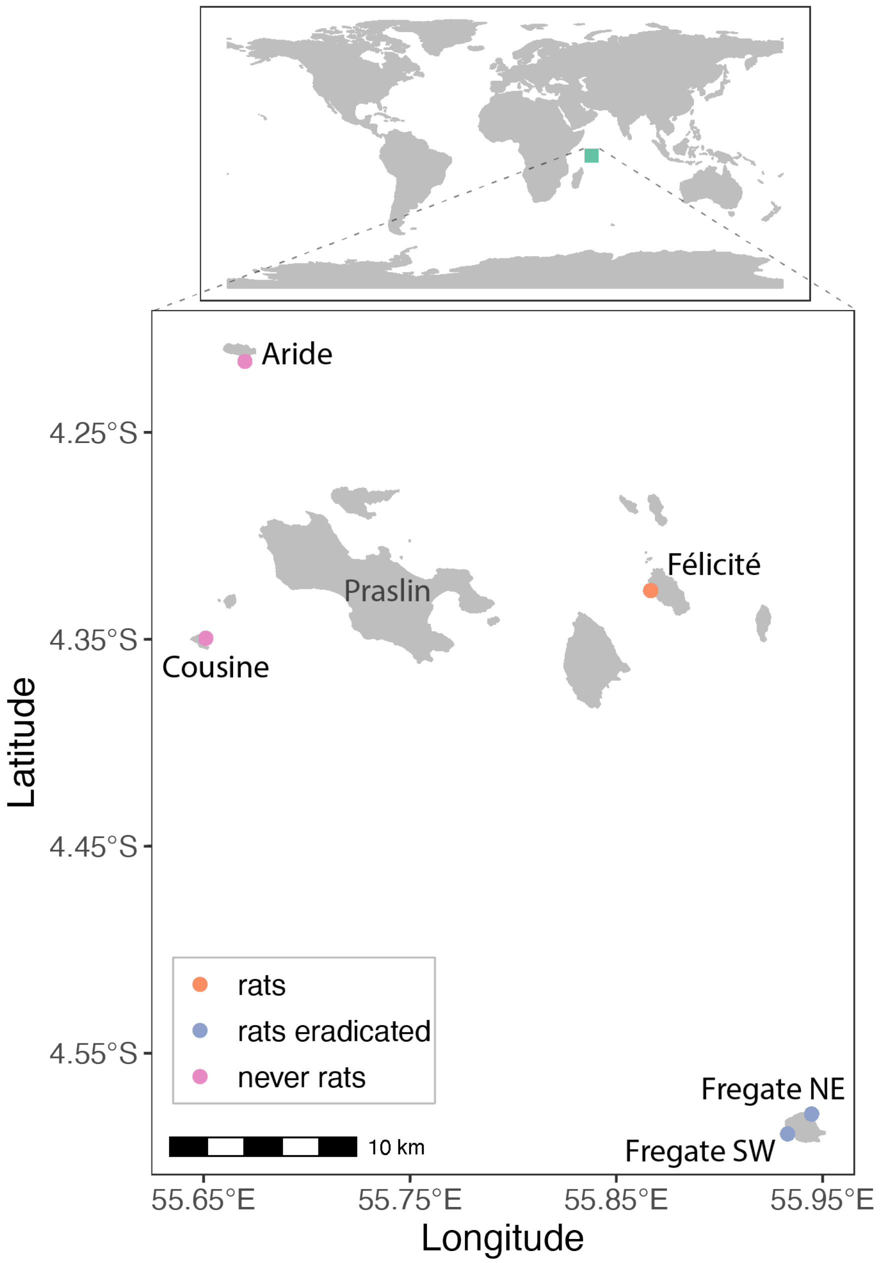

***S2 Fig*. Map of study sites, showing location on world map (top), and zoomed into the inner Seychelles (bottom).** Maps were created in R with associated packages *ggplot2*, *sf*, and *ggspatial*. Seychelles shapefile was obtained from https://data.humdata.org/dataset/cod-ab-syc under their CC-BY-IGO license.
