# Supplementary material for: Nutrient connectivity via seabirds enhances dynamic measures of coral reef ecosystem function
Source: PLoS Biol. 2025 Jul 8;23(7):e3003222. doi: 10.1371/journal.pbio.3003222 (PMC12237027; doi:10.1371/journal.pbio.3003222)
Supplement: S3 Fig — The study period (November 2022) is indicated by a thick orange border around the panel. The data underlying this figure are from the Seychelles International Airport provided by the Seychelles Meteorological Authority and can be found in https://doi.org/10.5281/zenodo.15485420. (PDF) [file pbio.3003222.s008.pdf]

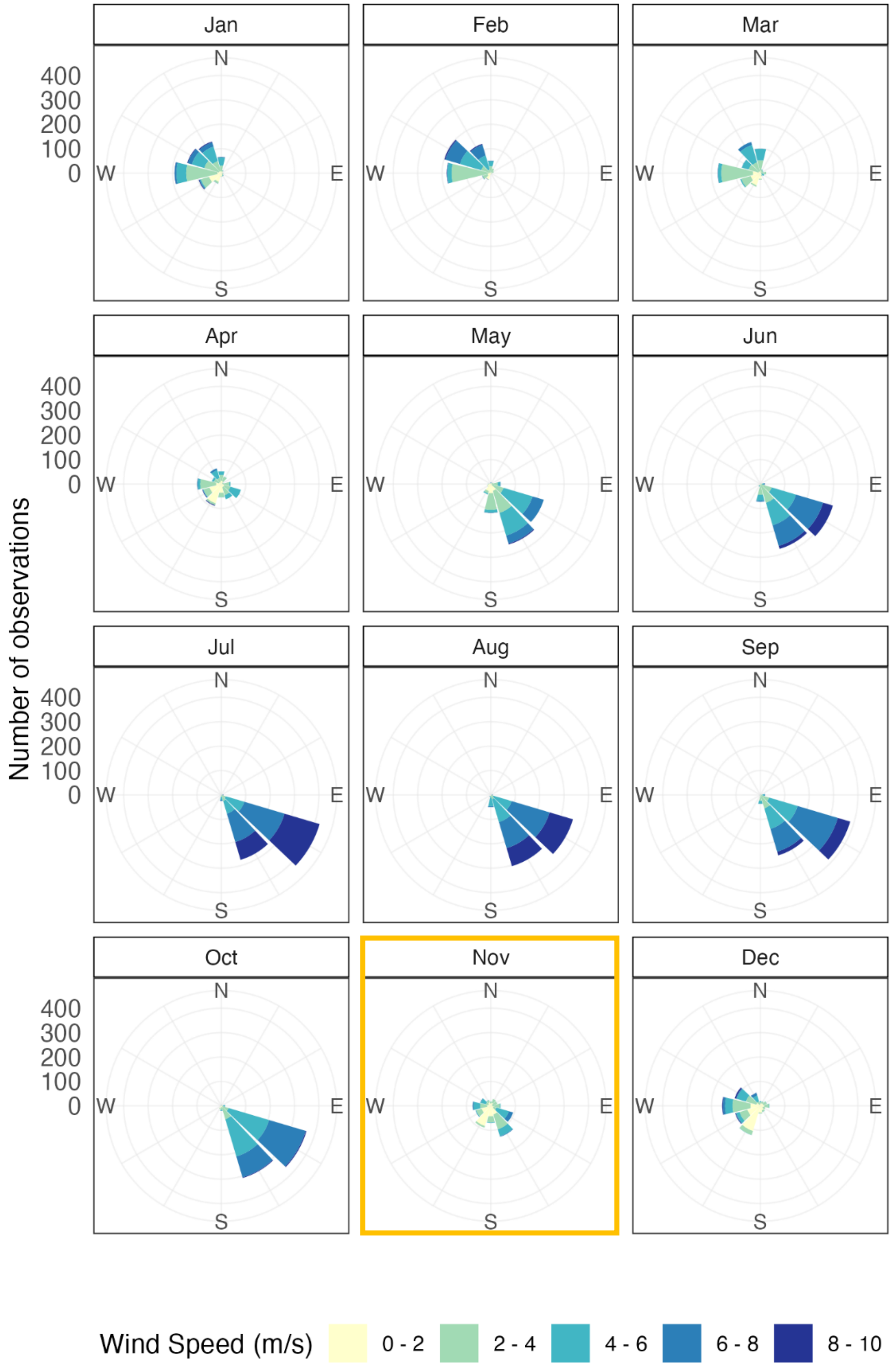

***S3 Fig*. Rose diagram of hourly wind direction and speed by month for 2022.** The study period (November 2022) is indicated by a thick orange border around the panel. The data underlying this figure are from the Seychelles International Airport provided by the Seychelles Meteorological Authority, and can be found in https://doi.org/10.5281/zenodo.15485420.
