# Supplementary material for: Nutrient connectivity via seabirds enhances dynamic measures of coral reef ecosystem function
Source: PLoS Biol. 2025 Jul 8;23(7):e3003222. doi: 10.1371/journal.pbio.3003222 (PMC12237027; doi:10.1371/journal.pbio.3003222)
Supplement: S4 Fig — Turf nutrients are the amount of seabird-derived nutrients in turf algae (measured as δ15N), turf productivity is growth (mm/day, measured in herbivore exclusion cages), turf cover is proportional cover along benthic transects (log-transformed), herbivore productivity is calculated from fish transects (log kg/ha/day), and herbivore biomass is from fish transects (log kg/ha). All metrics are site-level means. The data underlying this figure can be found in https://doi.org/10.5281/zenodo.15485420. (PDF) [file pbio.3003222.s009.pdf]

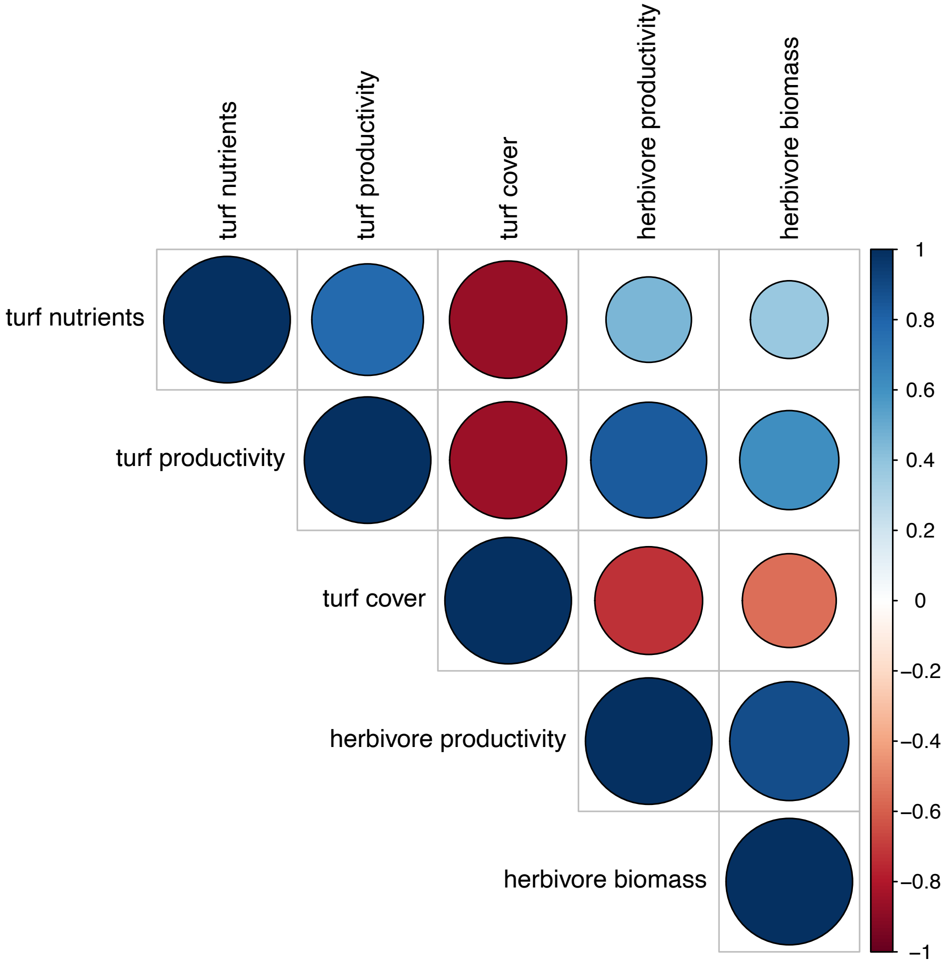

***S4 Fig*. Correlations among algal turf and herbivorous fish metrics.** Turf nutrients are the amount of seabird-derived nutrients in turf algae (measured as $\delta^{15}N$), turf productivity is growth (mm/day, measured in herbivore exclusion cages), turf cover is proportional cover along benthic transects (log-transformed), herbivore productivity is calculated from fish transects (log kg/ha/day), and herbivore biomass is from fish transects (log kg/ha). All metrics are site-level means. The data underlying this figure can be found in https://doi.org/10.5281/zenodo.15485420.
