# Supplementary material for: Nutrient connectivity via seabirds enhances dynamic measures of coral reef ecosystem function
Source: PLoS Biol. 2025 Jul 8;23(7):e3003222. doi: 10.1371/journal.pbio.3003222 (PMC12237027; doi:10.1371/journal.pbio.3003222)
Supplement: S5 Fig — (A, B) Herbivorous fish were grouped into functional groups based on how they feed, with community biomass in (A) and community productivity in (B). Colored circles represent transects, with the distance between points approximating community dissimilarity (i.e., points that are closer together have more similar communities, points that are farther apart have more different communities). Shaded areas represent minimum convex hull polygons for each site. Black triangles and text indicate the position of herbivore functional groups (A, B) and benthic groups (C). Gray arrows and text indicate the strength and direction of effects of environmental correlates. The data underlying this figure can be found in https://doi.org/10.5281/zenodo.15485420. (A) Turf productivity, turf nutrients, and structure were the environmental variables most associated with herbivorous fish community biomass (r2 = 0.52, 0.31, 0.26), and all showed positive associations with NMDS1. Turf-feeding croppers were the functional group most positively correlated with NMDS1 (cor = 0.76), and, thus, with these environmental variables, with macroalgal browsers and detritivorous brushers also showing positive correlations with NMDS1 (cor = 0.51, 0.53), while farming damselfishes showed a strong negative correlation with NMDS 1 (cor = −0.64). (B) Structure, turf productivity, and turf nutrients also showed strong associations with herbivorous fish community productivity (r2 = 0.47, 0.36, 0.26). Cropper productivity was the most associated with NMDS1 (cor = 0.86), with browsers also showing a strong positive association (cor = 0.72). Scrapers and farmers had the strongest negative associations with NMDS 1 (cor = −0.59, −0.54). (C) Exposure, turf nutrients, structure, and biomass of turf-removing herbivores were the environmental variables most associated with benthic communities (r2 = 0.69, 0.54, 0.34, 0.26), and all showed positive associations with NMDS1. Turf algae and sand/rubble were the benthic group [file pbio.3003222.s010.pdf]

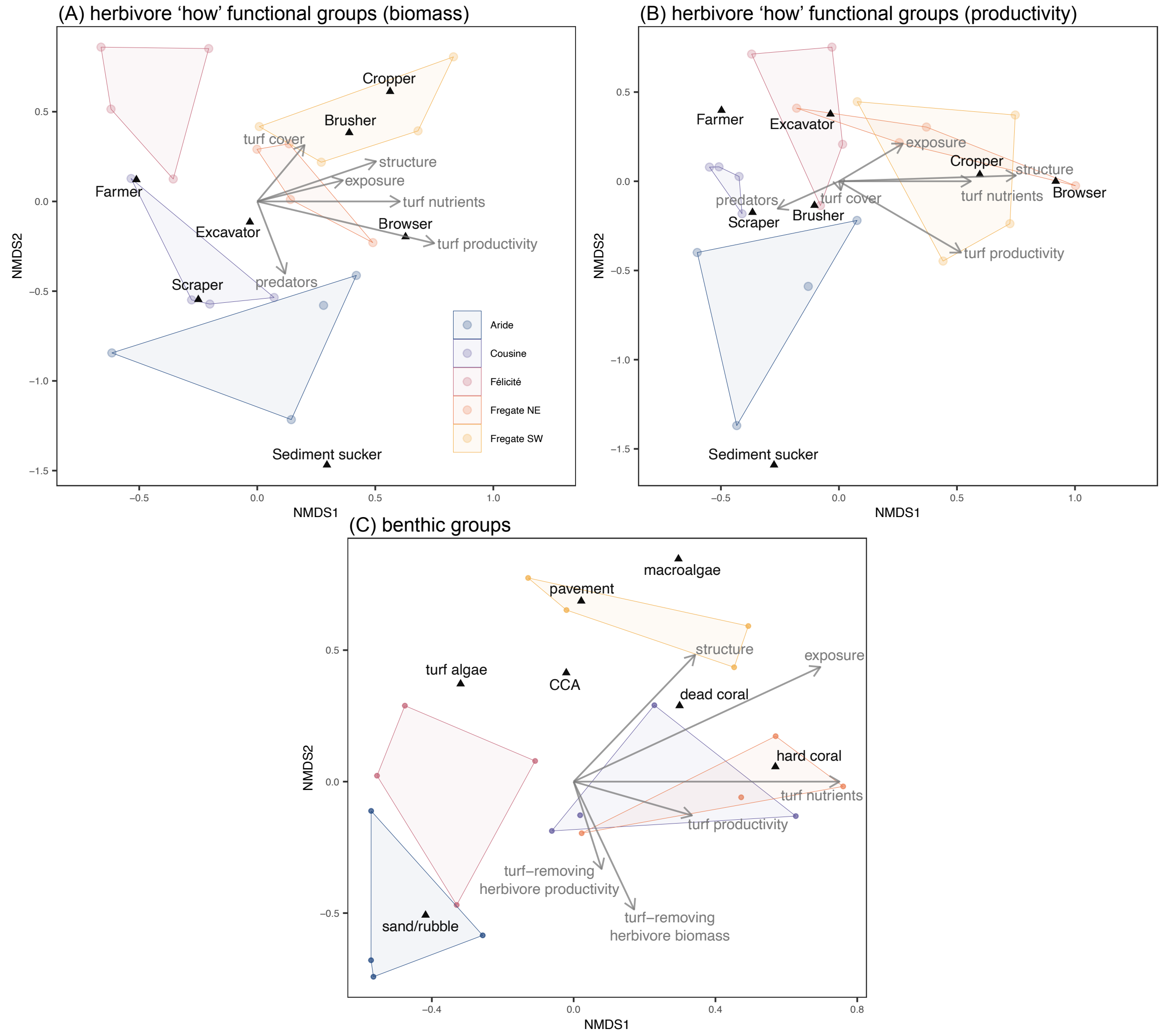

***S5 Fig*. Non-metric multidimensional scaling (NMDS) plots of herbivorous fish (A-B) and benthic (C) communities.** (A-B) Herbivorous fish were grouped into functional groups based on how they feed, with community biomass in (A) and community productivity in (B). Colored circles represent transects, with the distance between points approximating community dissimilarity (i.e., points that are closer together have more similar communities, points that are farther apart have more different communities). Shaded areas represent minimum convex hull polygons for each site. Black triangles and text indicate the position of herbivore functional groups (A-B) and benthic groups (C). Gray arrows and text indicate the strength and direction of effects of environmental correlates. The data underlying this figure can be found in https://doi.org/10.5281/zenodo.15485420.

(A) Turf productivity, turf nutrients, and structure were the environmental variables most associated with herbivorous fish community biomass ($r^2 = 0.52, 0.31, 0.26$), and all showed positive associations with NMDS1. Turf-feeding croppers were the functional group most positively correlated with NMDS1 (cor = 0.76), and thus with these environmental variables, with macroalgal browsers and detritivorous brushers also showing positive correlations with NMDS1 (cor = 0.51, 0.53), while farming damselfishes showed a strong negative correlation

with NMDS 1 (cor = -0.64). (B) Structure, turf productivity, and turf nutrients also showed strong associations with herbivorous fish community productivity (r^2 = 0.47, 0.36, 0.26). Cropper productivity was the most associated with NMDS1 (cor = 0.86), with browsers also showing a strong positive association (cor = 0.72). Scrapers and farmers had the strongest negative associations with NMDS 1 (cor = -0.59, -0.54). (C) Exposure, turf nutrients, structure, and biomass of turf-removing herbivores were the environmental variables most associated with benthic communities (r^2 = 0.69, 0.54, 0.34, 0.26), and all showed positive associations with NMDS1. Turf algae and sand/rubble were the benthic groups most negatively correlated with NMDS1 (cor = -0.36, -0.74, respectively), while hard coral was most positively correlated with NMDS1 (cor = 0.96).
