# Supplementary material for: Nutrient connectivity via seabirds enhances dynamic measures of coral reef ecosystem function
Source: PLoS Biol. 2025 Jul 8;23(7):e3003222. doi: 10.1371/journal.pbio.3003222 (PMC12237027; doi:10.1371/journal.pbio.3003222)
Supplement: S6 Fig — (a) Example herbivore exclusion cage one day after installation. Each cage was 14 × 14 × 10 cm with 1.2 cm openings and a 5-cm fringe around all sides through which nails were hammered into the substrate. (b) Example herbivore exclusion cage upon removal after 4–7 days. Turf algae grows quickly in the absence of herbivores, resulting in clear differences between the areas within the cage and immediately adjacent to the cage by the end of the experiment. (PDF) [file pbio.3003222.s011.pdf]

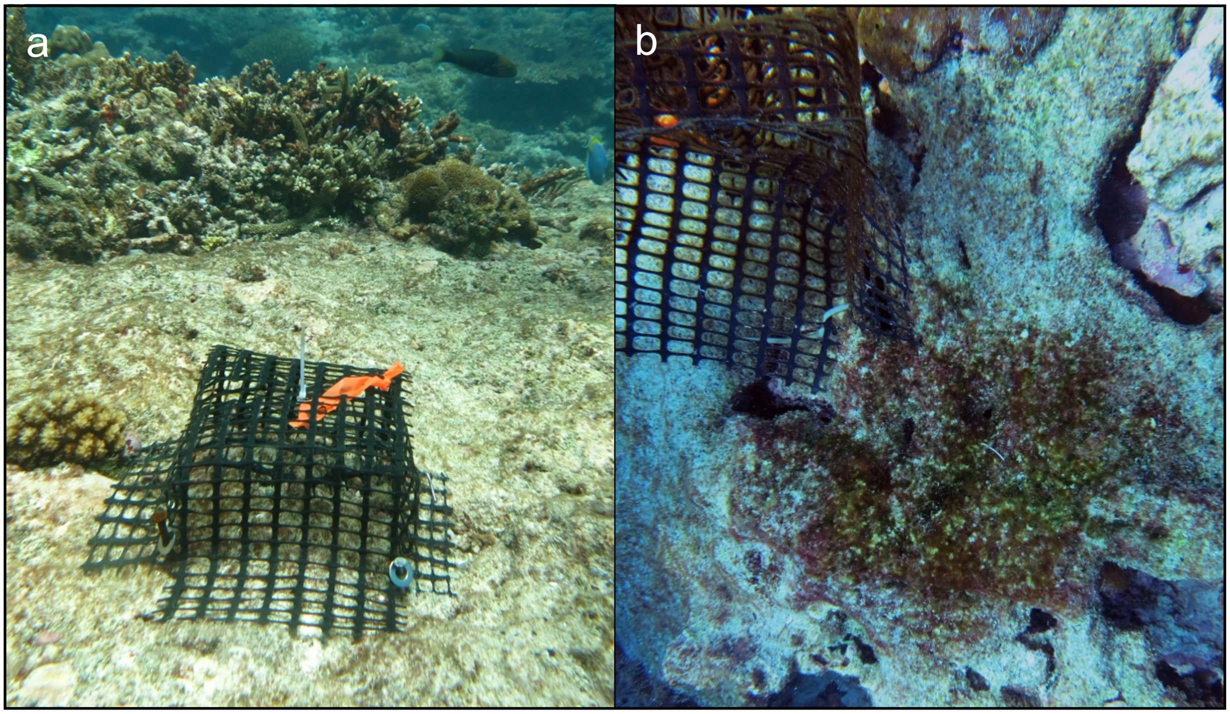

Figure S6. **Photographs of herbivore exclusion cages used for measurement of algal turf productivity.** (a) Example herbivore exclusion cage one day after installation. Each cage was 14 x 14 x 10 cm with 1.2 cm openings and a 5-cm fringe around all sides through which nails were hammered into the substrate. (b) Example herbivore exclusion cage upon removal after 4-7 days. Turf algae grows quickly in the absence of herbivores, resulting in clear differences between the area within the cage and immediately adjacent to the cage by the end of the experiment.
